# Cognitive Processing Speed across the Lifespan: Beyond the Influence of Motor Speed

**DOI:** 10.3389/fnagi.2017.00062

**Published:** 2017-03-22

**Authors:** Deena Ebaid, Sheila G. Crewther, Kirsty MacCalman, Alyse Brown, Daniel P. Crewther

**Affiliations:** Department of Psychology and Counselling, School of Psychology and Public Health, La Trobe UniversityMelbourne, VIC, Australia

**Keywords:** processing speed, aging, motor dexterity, motor speed, symbol search, coding, cognitive assessment, Inspection Time

## Abstract

Traditional neuropsychological measurement of cognitive processing speed with tasks such as the Symbol Search and Coding subsets of the WAIS-IV, consistently show decline with advancing age. This is potentially problematic with populations where deficits in motor performance are expected, i.e., in aging or stroke populations. Thus, the aim of the current study was to explore the contribution of hand motor speed to traditional paper-and-pencil measures of processing speed and to a simple computer-customized non-motor perception decision task, the Inspection Time (IT) task. Participants were 67 young university students aged between 18 and 29 (59 females), and 40 older adults aged between 40 and 81 (31 females) primarily with a similar education profile. As expected, results indicated that age group differences were highly significant on the motor dexterity, Symbol Search and Coding tasks. However, no significant differences or correlations were seen between age groups and the simple visual perception IT task. Furthermore, controlling for motor dexterity did not remove significant age-group differences on the paper-and-pencil measures. This demonstrates that although much of past research into cognitive decline with age is confounded by use of motor reaction times as the operational measure, significant age differences in cognitive processing also exist on more complex tasks. The implications of the results are crucial in the realm of aging research, and caution against the use of traditional WAIS tasks with a clinical population where motor speed may be compromised, as in stroke.

## Introduction

Cognitive processing speed when defined as the ability to process information rapidly, is closely related to the ability to perform higher-order cognitive tasks (Lichtenberger and Kaufman, 2012) and is often assumed to be the core issue responsible for deficits in performance on complex cognitive measures in aging populations (Salthouse, 1996; Salthouse and Ferrer-Caja, 2003). Neuropsychological testing has traditionally assessed processing speed across the lifespan with paper-and-pencil tests such as the Symbol Search and Coding tasks from the Information Processing Speed Index of the Wechsler Adult Intelligence Scale (WAIS) (Wechsler, 2008) i.e., Cornelis et al. (2014), Joy et al. (2004), and Kreiner and Ryan (2001). Such tests are reported to measure working memory, psychomotor and visuomotor processing speed, visual discrimination and attention; See Holdnack et al. (2013) and Wechsler (2008). However, despite the natural decline in motor speed and function with age, the extent that motor function affects age-related performance on such tasks has not been analyzed in detail.

Current theories of processing speed and age include the Sensory Deprivation hypothesis, the Common-Cause hypothesis, and the Information Degradation hypothesis. Though these theories may sometimes be considered as competing theories, they are relatively similar in suggesting a strong age induced interaction between declines in sensory function i.e., vision and audition, and a slowing in cognitive processing speed. A potential explanation for the link between sensory and cognitive decline was provided by Baltes and Lindenberger (1997) who concluded that sensory and cognitive function are both likely to be an expression of the “physiological architecture of the aging brain” (p. 13). Lindenberger and Baltes (1994) had previously reported that sensorimotor variables such as visual acuity, balance-gait, and auditory acuity, predicted 59% of total reliable variance in general intelligence. For a review on the interaction between perceptual and cognitive decline with aging, see Schneider and Pichora-Fuller (2000) and Roberts and Allen (2016) for a brief, more recent review. Considering that sensory function, particularly for hearing and vision is commonly reported to decline with age (for review see Fozard, 1990), the Sensory Deprivation hypothesis states that a lack of adequate sensory input over a prolonged period is likely to result in cognitive deterioration due to the prior neuronal atrophy (Oster, 1976; Valentijn et al., 2005). Similarly the Information Degradation hypothesis states that when perceptual signals are weakened or *degraded*, either due to experimental manipulations or age-related impaired perception, higher order cognitive processes are in turn affected (Schneider and Pichora-Fuller, 2000). This may be because the cognitive load is greater for weak perceptual signals, and thus require more cognitive resources to interpret the signal, which as a result, compromises cognitive performance (Zekveld et al., 2011). The major difference in the Sensory Deprivation hypothesis and the Common-Cause hypothesis lies in the interpretation as to when sensory and cognitive decline occur, with the Common-Cause hypothesis suggesting concurrent peripheral and central decline occurring simultaneously with declines in aspects of cognition, such as memory and processing speed (Fozard, 1990; Lindenberger and Baltes, 1994). This may be indicative of common declining attention and efficiency of all the neuronal networks in the brain as well as the eyes. Variations in interpretation are often dependent on neuroanatomical explanations of processing speed decline across the lifespan; a realm on which an abundance of research exists (Chee et al., 2009; Bendlin et al., 2010; Fjell and Walhovd, 2010; Eckert, 2011; Kerchner et al., 2012; Hong et al., 2015).

Addressing the potential confound of motor performance on paper-and-pencil tasks designed to measure cognitive processing speed, Crowe et al. (1999) investigated cognitive determinants of performance on the Digit Symbol-Coding Test (Coding) and Symbol Search, with particular attention to motor execution. It was concluded that motor speed was a significant predictor of scores, highlighting the large contribution of motor ability to variance in performance on these paper-and-pencil measures of processing speed. Kreiner and Ryan (2001) also examined the contribution of motor skill versus memory ability to scores on the Digit Symbol-Coding subtest of the WAIS-III in a clinical sample of patients with substance abuse, depression, or post-traumatic stress disorder and concluded that hand motor skill explained a large portion of the variance in Digit Symbol-Coding variance, whereas memory ability explained little additional variance in Digit Symbol Copy variance when motor skill scores were controlled. Similar results were reported by Joy et al. (2000), in a further clinical sample of patients with diagnoses such as alcoholism and other substance abuse, depression, and post-traumatic stress disorder, emphasizing that motor skill may be a more important contributor than cognitive performance to scores on traditional paper-and-pencil tasks in the WAIS.

An alternative task, though not commonly used clinically, or even experimentally in older participants, is computer based measures of processing speed that do not require a motor response. The simplest of such tasks is the Inspection Time as a measure of perceptual processing speed. Inspection Time (IT; Vickers et al., 1972) is a psychophysical computer-based task that has often been reported to measure time required to assess perceptual thresholds, without reliance on manual dexterity (Deary and Stough, 1996) or even eye movements. Indeed, Gregory et al. (2008) have found that momentary changes in performance on IT predicts future performance on cognitive tests measuring perceptual speed, working memory, and fluid reasoning, and may also serve as a biomarker for cognitive decline (Nettelbeck and Wilson, 2004, 2005).

The current study aimed to compare the extent to which performance on traditional measures of cognitive processing speed (i.e., the Symbol Search and Coding) are confounded by motor speed in young and older adults. It was hypothesized that younger adults would outperform older adults on the Symbol Search and Coding tasks but not the IT task, and not when controlling for motor dexterity. It was also hypothesized that the Symbol Search and Coding tasks would be significantly correlated with motor dexterity (i.e., Pegboard scores).

## Methods

### Sample

The present study included two age groups: 67 young adults (Females = 59, *M* age = 19.6, age range: 18–29), and 40 healthy older adults (Females = 31, *M* age = 66.7, age range: 40–81). The young adult group predominantly consisted of first year Psychology students, who received course credit, while the older adult group were predominantly recruited through personal networks or recruited through the University of the Third Age (U3A), and received a $20 Coles-Myer voucher. Exclusion criteria included previous diagnosis of a neurological disorder and the inability to understand and speak English with basic competence. A demographic questionnaire elicited information on age, lifestyle status, and education level. A measure of general negative affect: The Depression Anxiety and Stress Scale (DASS-21; Lovibond and Lovibond, 1995) was administered as a screening tool.

### Materials

#### Purdue pegboard

The three subtasks of the Purdue Pegboard (Reddon et al., 1988) were used as a measure of fine motor dexterity, specifically, finger dexterity (Tiffin and Asher, 1948). Performance on the Pegboard has previously been reported to decline with advanced age (Desrosiers et al., 1995). The Purdue Pegboard comprises a wooden board with two parallel rows of 25 holes each, into which as many cylindrical metal pegs (1 mm in diameter and 25 mm in length) are sequentially moved from a groove at the top of the board and placed in one of the holes by the participant in 30 s. Three sets of trials (dominant hand, then the non-dominant hand, and finally bimanually) were completed after instructions. The score is the number of pins successfully placed. In the current study, individual scores across all three conditions of the pegboard were used.

#### Symbol search and coding (WAIS-IV)

The Symbol Search and Coding subtests from the WAIS-IV were administered for 2 min each as per the WAIS-IV kit instructions.

During Symbol Search, two target symbols appearing on the left of a row are sought among an array of five symbols on the right. The individual responds by either marking the identical symbol, or a “no” box (if the matching symbol is not present in the array) with a pencil. Performance was measured as the number of symbols accurately identified in 2 min. In healthy adults, raw scores on Symbol Search have been shown to decline by more than 50% between the ages of 25 and 65 (Wechsler, 2008). However, this measure is still commonly used as an indicator of processing speed without accounting for possible age related decline that is often associated with healthy aging (Seidler et al., 2002, 2010).

The Coding task requires an individual to copy (with a pencil) the appropriate symbol in a box underneath a digit (one-to-nine), using a key at the top of the page containing digits and their corresponding symbols. Performance is based on the number of pairs correctly copied in 2 min.

#### Modified inspection time

**Figure 1:**
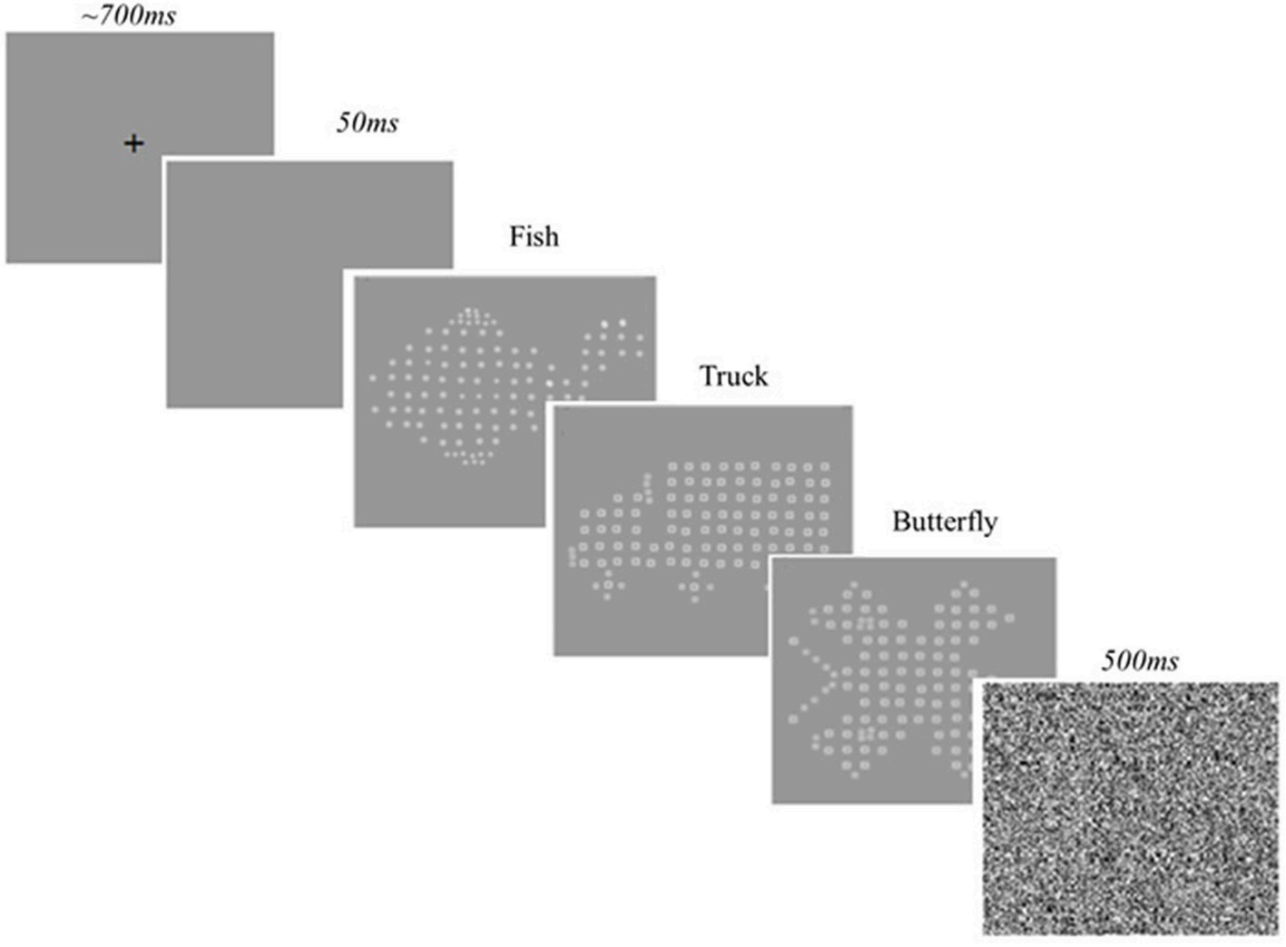
**Modified Inspection Time task trial**. Only one target stimuli of fish, truck, or butterfly is presented per trial.

A modified IT task, based on the version of Vickers et al. (1972) was adapted by Brown and Crewther (2015) and programmed using the psychophysics program, Vpixx (www.Vpixx.com). Stimuli were presented on an Apple eMac Computer running at 89 Hz screen refresh rate. Across the trials, a fixation cross was presented for a random duration between 700 and 1,000 ms, followed by a blank screen for 50 ms, then the target stimulus was presented at variable exposure times beginning at 1 s, immediately followed by a mask, presented for 500 ms. These exposure times were not altered between the various age groups. Target stimulus was either a *fish, truck*, or *butterfly* (See Figure 1). Participants were required to indicate which target stimulus they had been presented by pressing the appropriate one of three buttons on the keyboard. A response triggered the start of the next trial. The task employs an inbuilt Visual Parameter Estimation by Sequential Testing (VPEST) algorithm, designed to estimate the exposure threshold required to discriminate and identify which of the three possible stimuli was presented: fish, truck or butterfly. Confidence intervals and estimations of exposure time were calculated as part of the Vpixx program. A lower estimated exposure time indicated a faster cognitive processing speed threshold (specifically, perceptual speed). See Figure 1 for an example trial of IT task.

### Procedure

All participants were guided through the tests outlined above and all tasks were preceded by practice trials. Total testing time was approximately 1 h. Practice trials for each condition on the Purdue Pegboard were usually only done once, unless the participant requested more. For the Symbol Search task, three arrays of untimed practice items were completed prior to attempting the test. Similarly, for the Coding task, there were nine untimed practice trials. The IT task was also preceded with five practice trials, or until the participant was satisfied they did not need more practice. To ensure accurate results for the IT, the task was re-done if the confidence interval as indicated by the Vpixx data output was below 80%.

### Data analysis

An independent samples *t*-test was used to compare performance on dependent measures between age-groups. This was followed by an Analysis of Covariance (ANCOVA) aimed at exploring performance on dependent measures between age-groups while controlling for motor dexterity. An independent samples *t*-test on IT performance between age-groups was also conducted to explore performance between young and older adults on all dependent measures. To explore the relationship between the Symbol Search and Coding with motor dexterity, correlation analyses were conducted between Pegboard scores and Symbol Search and Coding tasks. A second analysis was then conducted after splitting participants into three age groups, namely, young adults aged 18–29 (*N* = 67), middle-aged adults aged 40–59 (*N* = 9), and older adults aged 61–81 (*N* = 31). Though we acknowledge that the small numbers in the middle-aged group is a limitation to an ANOVA, analyses were conducted to determine whether any absence of significance using two age groups was due to the nine participants in the intermediate age-group (i.e., middle-age group).

#### Analyses of results using two age groups (young and older adults)

##### Preliminary analysis of results between education levels in age groups

Given the various levels of education within the sample, an ANOVA was conducted to determine whether performance on dependent measures (i.e., IT, Symbol Search, Coding, and Pegboard) were significantly different between participants “highest level of completed education” within young and older adults. See Table 1A for descriptive statistics of highest level of completed education for young and older adults. Results of the ANOVA indicate that there were no significant differences between participants' highest level of completed education and performance on dependent measures in both the young and older adult groups. See Tables 1B,C.

**Table 1A:** **Highest level of completed education in young and older adults**.

**Highest level of completed education**	**Younger adults %**	**Older adults %**
Secondary school	91	27.5
Diploma	4.5	20
Bachelor degree	4.5	20
Post-graduate degree	0	17.5
Masters	0	12.5
Doctorate/PhD	0	2.5

**Table 1B:** **Analysis of variance (ANOVA) between highest level of completed education and performance on dependent measures in young adults**.

**Variable**	**Sum of squares**	***df***	**Mean square**	***F***	***p***	**Partial η^2^**
SymS	135.039	66	67.520	1.148	0.324	0.035
Cod	323.055	66	161.527	0.941	0.396	0.029
IT	0.147	61	0.073	2.715	0.074	0.084
Pgb.dom	1.868	66	0.934	0.265	0.768	0.008
Pgb.non	1.235	66	0.617	0.236	0.790	0.007
Pgb.2	48.865	66	24.432	1.642	0.202	0.049

**Table 1C:** **Analysis of variance (ANOVA) between highest level of completed education and performance on dependent measures in older adults**.

**Variable**	**Sum of Squares**	***df***	**Mean Square**	***F***	***p***	**Partial η^2^**
SymS	386.487	39	77.297	1.601	0.186	0.191
Cod	1073.241	39	214.648	1.271	0.299	0.157
IT	0.40	32	0.008	1.506	0.216	0.190
Pgb.dom	4.829	39	0.966	0.197	0.962	0.028
Pgb.non	5.402	39	1.080	0.307	0.905	0.043
Pgb.2	113.860	39	22.772	1.257	0.305	0.156

##### Preliminary analysis of results between genders

Given the uneven spread of genders within our sample (i.e., 90 Females, 17 Males), an independent samples *t*-test was conducted to determine whether there were differences in performance on any dependent measures within each age group across genders.

Results revealed no significant differences on any dependent measure between genders in the younger group. However, results demonstrated significant difference for Coding performance between genders for the older adults showing that males outperformed females, *M* difference = 11.66, *t*_(38)_ = 2.478 *p* = 0.018. See Table 2.

**Table 2:** **Mean difference between genders and scores on dependent measures in young and older adults**.

	**Younger adults**					**Older adults**				
**Variable**	***t***	***df***	**Mean difference**	**95% C.I. Mean difference**	**η2**	***t***	***df***	**Mean difference**	**95% C.I. Mean difference**	**η2**
SymS	0.326	65	0.951	−4.87–6.78	0.002	−1.11	38	−3.02	−8.53–2.49	0.031
Cod	−0.626	65	−3.10	−12.99–6.794	0.006	2.478^*^	38	11.66	2.14–21.184	0.139
IT	1.515	60	0.109	−0.035–0.253	0.037	0.709	36	0.02	−0.040–0.083	0.014
Pgb.dom	−0.216	65	−0.153	−1.56–1.26	0.001	−0.890	38	−0.71	−2.32–0.90	0.020
Pgb.non	0.832	65	0.502	−0.70–1.71	0.011	−0.983	38	−0.67	−2.04–0.707	0.025
Pgb.2	−1.152	65	−1.68	−4.61–1.24	0.020	−1.381	38	−2.24		0.048

*SymS, Symbol Search; Coding, Coding; IT, Inspection Time; Pgb.dom, Pegboard (dominant hand condition); Pgb.non, Pegboard (non-dominant hand condition); Pgb.2, Pegboard (both hands condition)*.

* *p ≤ 0.05*.

## Results

Table 3 depicts demographic information, i.e., age and education of the sample.

**Table 3:** **Descriptive statistics for age and years of education for younger and older adults**.

	**Younger adults**				**Older adults**			
**Variable**	***N***	***M***	***SD***	**Range**	***N***	***M***	***SD***	**Range**
Age	67	19.64	2.26	18–29	40	66.7	9.72	40–81
YrsEdu	67	7.64	1.06	7–11	40	10.4	3.37	4–21

*Age, age in years; YrsEdu, years of education since Year 7 (start of high school)*.

Sample size, means, standard deviations and range for each of the variables by age-group are shown in Table 4.

**Table 4:** **Descriptive statistics for each dependent variable**.

	**Younger adults**				**Older adults**			
**Variable**	***N***	***M***	***SD***	**Range**	***N***	***M***	***SD***	**Range**
SymS	67	39.537	7.688	24–60	40	28.450	7.211	12–48
Cod	67	78.731	13.090	37–121	40	54.075	13.212	30–91
IT	62	0.077	0.169	0.008–1.028	38	0.124	0.075	0.016–0.343
Pgb.dom	67	16.134	1.858	11–20	40	13.550	2.099	9–18
Pgb.non	67	14.433	1.598	11–18	40	12.850	1.791	9–16
Pgb.2	67	23.99	3.894	9–31	40	20.400	4.325	7–31

*SymS, Symbol Search; Coding, Coding; IT, Inspection Time; Pgb.dom, Pegboard (dominant hand condition); Pgb.non, Pegboard (non-dominant hand condition); Pgb.2, Pegboard (both hands condition)*.

### Relationships among age and dependent measures

Correlational analyses were performed to investigate the strength, direction and significance of associations between age and dependent measures of motor dexterity and processing speed. Results revealed significant moderate to strong positive correlations with increasing age and scores on all three conditions of the Pegboard, indicating age-related motor dexterity decline. Significant strong negative correlations were also demonstrated between increasing age and scores on the Symbol Search and Coding tasks, indicating a slower processing speed as age increases. A significant weak correlation was also exhibited between increasing age and higher scores on the IT, indicating that a longer stimulus exposure duration was required to identify a visual stimulus as age increased.

### Relationships between motor dexterity and measures of processing speed

Correlational analyses were performed to investigate the strength, direction and significance of associations between the Pegboard and measures of processing speed, namely, Symbol Search, Coding, and IT. Results demonstrated significant positive correlations between each condition of the Pegboard and both paper-and pencil measures of Processing speed, i.e., the Symbol Search and Coding. This indicated that better motor dexterity was associated with a faster processing speed, as measured by the Symbol Search and Coding. Contrary to this, there was no significant relationship between any condition of the Pegboard and performance scores on the IT, indicating that motor dexterity is unlikely to impede or improve performance on the IT. A full correlation table of all measures is shown in Table 5.

**Table 5:** **Pearson product-moment correlations between dependent measures**.

**Measure**	**Age**	**SymS**	**Coding**	**IT**	**Pgb.dom**	**Pgb.non**	**Pgb.2**
1. Age	–						
2. SymS	−0.613^**^	–					
3. Coding	−0.673^**^	0.671^**^	–				
4. IT	0.208^*^	−0.081	−0.086	–			
5. Pgb.dom	−0.603^**^	0.439^**^	0.440^**^	−0.093	–		
6. Pgb.non	−0.496^**^	0.454^**^	0.434^**^	0.034	0.640^**^	–	
7. Pgb.2	−0.433^**^	0.376^**^	0.335^**^	−0.193	0.560^**^	0.619^**^	–

*SymS, Symbol Search; Coding, Coding; IT, Inspection Time; Pgb.dom, Pegboard (dominant hand condition); Pgb.non, Pegboard (non-dominant hand condition); Pgb.2, Pegboard (both hands condition)*.

** *p ≤ 0.01*,

* *p ≤ 0.05*.

### Differences in performance on dependent measures by age-group

An Independent-samples *t*-test was conducted to compare performance on dependent measures between younger and older adults. Significant age-group differences were found for mean scores on all paper-and-pencil measures, namely, Symbol Search and Coding, as well as on all Pegboard tasks. However, significant age group differences were not demonstrated for performance on psychophysical measure of processing speed, i.e., the IT task.

The results of the independent-samples *t*-tests are shown in Table 6.

**Table 6:** **Mean difference between age group and scores on dependent measures**.

**Variable**	***t***	***df***	**Mean difference**	**95% C.I. Mean difference**	**η^2^**
SymS	7.385^**^	105	11.087	8.110–14.064	0.342
Cod	9.392^**^	105	24.656	19.45–29.861	0.457
IT	−1.618	98	−0.047	−0.104–0.010	0.026
Pgb.dom	6.629^**^	105	2.584	1.811–3.357	0.295
Pgb.non	4.738^**^	105	1.58	0.920–2.245	0.176
Pgb.2	4.419^**^	105	3.58	1.977–5.193	0.157

*SymS, Symbol Search; Coding, Coding; IT, Inspection Time; Pgb.dom, Pegboard (dominant hand condition); Pgb.non, Pegboard (non-dominant hand condition); Pgb.2, Pegboard (both hands condition)*.

** *p < 0.01*.

### Partial correlation controlling for motor dexterity

An ANCOVA was conducted to explore the relationship between age and outcome on processing speed measures (i.e., Symbol Search, Coding, and IT) while controlling for motor dexterity of the dominant hand, given that participants used their dominant hand to complete the paper-and-pencil tasks used in the current study. Despite controlling for motor dexterity, age still had a large significant effect on Symbol Search scores *F*_(1, 104)_ = 27.751, *p* < 0.01, partial η^2^ = 0.211 and on Coding scores *F*_(1, 104)_ = 52.464, *p* < 0.01, partial η^2^ = 0.335, and thus, there were still significant differences on paper-and-pencil task scores (i.e., Symbol Search and Coding) between young and older adults, even when controlling for motor dexterity.

An inspection of zero order correlation revealed that controlling for motor dexterity influenced the strength of the relationship between age and Symbol Search scores (*r* = −0.466) and age and Coding scores (*r* = −0.573). However, controlling for motor dexterity had little impact on the strength of the relationship between age and IT scores (*r* = 0.190). See Table 7 for Analysis of Covariance table between age and Symbol Search and Coding while controlling for motor dexterity.

**Table 7:** **Analysis of covariance (ANCOVA) between age and performance on paper-and-pencil tasks while controlling for motor dexterity**.

**Variable**	**Sum of squares**	***df***	**Mean square**	***F***	***p***	**Partial η^2^**
SymS	3099.43	106	1549.72	27.28	<0.01	0.344
Cod	15298.25	106	7649.12	44.07	<0.01	0.459
IT	0.071	99	0.036	1.79	0.174	0.035
Pgb.dom	224.92	106	112.46	34.19	<0.01	0.397
Pgb.non	111.03	106	55.51	22.54	<0.01	0.312
Pgb.2	407.28	106	203.64	12.87	<0.01	0.198

*SymS, Symbol Search; Coding, Coding; IT, Inspection Time; Pgb.dom, Pegboard (dominant hand condition); Pgb.non, Pegboard (non-dominant hand condition); Pgb.2, Pegboard (both hands condition)*.

### Analyses of results using three age groups (young, middle-aged and older adults)

#### Mean differences in performance on dependent measures by three age groups

To ensure that the nine participants aged between 40 and 59 did not bias results when using only two age groups, analyses were run using three age groups. Specifically, the sample was divided into young adults aged 18–29 (*N* = 67), middle-aged adults aged 40–59 (*N* = 9), and older adults aged 61–81 (*N* = 31). An ANOVA was conducted to assess mean differences in performance on dependent measures by age-group. Results demonstrated significant age-group differences for mean scores on all paper-and-pencil measures (i.e., Symbol Search and Coding), as well as on all conditions of the Pegboard task. However, significant age-group differences were not demonstrated for performance on the IT task. These results coincide with results found with the sample divided into two age groups (i.e., young adults aged 18–29 and older adults aged 40–81). Results of the ANOVA are shown in Table 8.

**Table 8:** **Analysis of variance (ANOVA) between age group and performance on dependent measures**.

**Variable**	**Type III sum of squares**	***df***	**Mean square**	**F**	***p***	**Partial η^2^**
SymS	1531.735	104	1531.735	27.751	<0.01	0.211
Cod	9014.634	104	9014.634	52.464	<0.01	0.335

*SymS, Symbol Search; Coding, Coding*.

#### Analyses of results between genders within three age groups

An analysis of results between genders within each age groups was also conducted with the sample divided into three age-groups. An independent samples *t*-test was conducted to determine whether there were differences in performance on any dependent measures within each age group across genders.

Results revealed no significant differences on any dependent measure between genders in the younger group, as well as in the middle-aged group. However, results again demonstrated significant differences in Coding performance between genders for the older adults with males outperforming females, *M* difference = 12.46, *t*_(29)_ = 2.485 *p* = 0.019. See Table 9. These results also mirror those demonstrated with the sample divided into two groups (i.e., young and older adults).

**Table 9:** **Mean difference between genders and scores on dependent measures in young, middle-aged, and older adults**.

	**Younger adults**					**Middle-aged adults**					**Older adults**				
**Variable**	***t***	***df***	**Mean difference**	**95% C.I. Mean difference**	**η^2^**	***t***	***df***	**Mean difference**	**95% C.I. Mean difference**	**η^2^**	***t***	***df***	**Mean difference**	**95% C.I. Mean difference**	**η^2^**
SymS	0.326	65	0.951	−4.873–6.775	0.002	0.130	7	0.929	−15.941–17.798	0.002	−1.409	29	−4.143	−10.157–1.871	0.064
Cod	−0.626	65	−3.102	−12.997–6.794	0.006	0.689	7	8.929	−21.711–39.568	0.064	2.485^*^	29	12.458	2.205–22.712	0.176
IT	1.515	60	0.109	−0.0349–0.253	0.037	0.475	7	0.011	−0.0452–0.068	0.031	0.688	27	0.026	−0.051–0.102	0.017
Pgb.dom	−0.216	65	−0.153	−1.560–1.255	0.001	−0.350	7	−0.357	−2.772–2.058	0.017	−1.004	29	−0.798	−2.4230–0.8278	0.034
Pgb.non	0.832	65	0.502	−0.703–1.707	0.011	0.143	7	0.143	−2.218–2.504	0.003	−1.416	29	−0.887	−2.168–0.394	0.065
Pgb.2	−1.152	65	−1.686	−4.609–1.237	0.020	−0.557	7	−1.429	−7.497–4.639	0.042	−1.327	29	−2.452	−6.232–1.327	0.057

*SymS, Symbol Search; Coding, Coding; IT, Inspection Time; Pgb.dom, Pegboard (dominant hand condition); Pgb.non, Pegboard (non-dominant hand condition); Pgb.2, Pegboard (both hands condition)*.

* *p ≤ 0.05*.

#### Partial correlation controling for motor dexterity with three age groups

An ANCOVA was conducted to explore the relationship between age and outcome on processing speed measures (i.e., Symbol Search, Coding, and IT) while controlling for motor dexterity of the dominant hand. Despite controlling for motor dexterity, age still had a large significant effect on Symbol Search scores *F*_(1, 106)_ = 13.748, *p* < 0.01, partial η^2^ = 0.344 and on Coding scores *F*_(1, 106)_ = 26.009, *p* < 0.01, partial η^2^ = 0.459, and thus, there were still significant differences on paper-and-pencil task scores (i.e., Symbol Search and Coding) between young, middle-aged, and older adults, even when controlling for motor dexterity. Results of the ANCOVA are presented in Table 10.

**Table 10:** **Analysis of covariance (ANCOVA) between age and performance on paper-and-pencil tasks while controlling for motor dexterity**.

**Variable**	**Type III sum of squares**	***df***	**Mean square**	***F***	***p***	**Partial η^2^**
SymS	1532.283	106	766.141	13.748	<0.01	0.344
Cod	9021.356	106	4510.678	26.009	<0.01	0.459

*SymS, Symbol Search; Coding, Coding*.

## Discussion

The most important findings of this study are that although motor reaction times were correlated with age, decline in cognitive processing speed across the lifespan is not fully/completely explained by motor performance. This is the case whether using traditional paper-and-pencil measures from the WAIS-IV; or IT, a non-motor measure of visual perceptual processing speed.

### Age and performance on measures of processing speed

As expected, significant age group differences were demonstrated on the Symbol Search and Coding tasks but not on the Inspection Time task. Declines in performance on the Symbol Search and Coding demonstrated in the current study are in line with the body of evidence referenced earlier that indicates processing speed declines with age (Cerella and Hale, 1994; Salthouse, 1996; Holdnack et al., 2013; Hong et al., 2015; Tam et al., 2015). Furthermore, findings are consistent with research indicating that age related processing slowing is reflected on tasks such as the Symbol Search and Coding (Gilmore et al., 2006), where declines in performance are generally most prominent from the age of 45 (Hoyer et al., 2004). Findings from the current study are in line with, and may be explained by current theories of processing speed decline across the lifespan, though this study cannot determine the temporal relationship between sensory processing and neural networks.

Results from the current study are also consistent with those found in a study conducted by Gilmore et al. (2006) who used multiple forms of a symbol-digit substitution task (similar to the Symbol Search and Coding used in the current study), where significant differences in performance between young and older adults were demonstrated. Interestingly however, when visual contrast sensitivity deficit of older adults was simulated on tasks by applying a digital filter, this markedly reduced the number of items that younger participants could complete on the coding task. Gilmore et al. (2006) concluded that contrast sensitivity deficits were linked to visual search speed, affecting overall performance on cognitive tasks, further lending support to the *Information Degradation hypothesis, Sensory Deprivation hypothesis, and Common-Cause hypotheses*. In line with these findings, various longitudinal studies have demonstrated that impairments in sensory perception are progressively associated with cognitive decline. For example, Lin et al. (2004) and Lin et al. (2013) reported that women with impaired vision or hearing had a faster rate of cognitive decline over a 4 or 6 year period compared to those without sensory impairment An explanation for this is provided by Rabbitt (1991) who suggested that an increased effort was necessary to recognize words when perceptual signals are weak, as the extra effort encoding or interpreting the degraded sensory signal commands cognitive resources that would otherwise be used in encoding and rehearsal. In turn, this results in a slower overall processing speed. Zekveld et al. (2011), also found this using pupillometry to demonstrate the effects of varying strengths of perceptual signals (i.e., verbal sentence intelligibility) in older adults with and without hearing loss.

Although significant age related group differences for performance on IT were not demonstrated in the current study, IT performance did correlate with age within groups, and this is in line with research suggesting that IT performance declines with increased age (Nettelbeck and Rabbitt, 1992; Gregory et al., 2008). Interestingly IT has been demonstrated to account for 25% of the variance in human intelligence (Deary and Stough, 1996). With this in mind, it may be the case that the results from the current study that showed no significant differences between age-groups and threshold times on the IT, may be partly due to the highly-educated sample across both age groups. Indeed, the majority of participants were university educated, and over 30% of the older participants held post-graduate academic degrees. It may be the case then, that IT is a more accurate representation of cognitive performance in similarly educated populations, a suggestion made by Deary et al. (2010) who also propose that the IT may be an effective biomarker of cognitive aging. It is also apparent that the IT is a quick, useful test for aging and health research.

### Motor dexterity and its relationship with age and processing speed measures

As expected, motor dexterity on the Purdue Pegboard was significantly negatively correlated with age (Seidler et al., 2002, 2010), an observation prominent particularly for participants aged over 65 (Carmeli et al., 2003; Murata et al., 2010). Furthermore, and contrary to expectations, even when motor dexterity on the Purdue Board was controlled, Symbol Search and Coding still correlated with age. This indicated that older participants still performed worse on the Symbol Search and Coding tasks that are designed to measure cognitive processing speed, short-term visual memory, visual-motor coordination, visual discrimination, speed of mental operation, attention, concentration, as well as psychomotor speed (Wechsler, 2008) even when motor dexterity was statistically controlled.

### Limitations

The populations used were both limitations and strengths of the current study, as it is unusual to include similarly educated young and older groups given the commonly reported link between education and cognitive performance (Stern, 2002; Zahodne et al., 2011), and association with higher IQ scores in later life. Samples varying in education levels may be of interest in future research assessing similar cognitive constructs. The uneven sex distribution of the sample was a further limitation of the study. As there were significant differences on performance scores on the Coding task between genders in the older adults, this relationship may be of interest for future research. Furthermore, to avoid any confound of genders on performance scores on similar cognitive tasks, an even spread of genders in a sample may ensure a more robust design for future research. The sample sizes of the experimental groups also serve as a limitation in the current study especially in regards to statistical analyses of the middle-aged group. To ensure robustness of analyses and results, future research investigating similar constructs should aim to have an even number of participants in the various age groups.

## Conclusions and future direction

The current study aimed to explore the issue of whether traditional measures of cognitive processing speed such as the Symbol Search and Coding Subsets of the WAIS-IV accurately reflect cognitive processing speed, or whether they are confounded by motor speed in older participants. Thus, to our knowledge the current study was one of the first to explicitly explore the issue of motor confounds and age and extract the value of the WAIS subtests as measures of complex cognition in comparison to the Simple IT task in groups of similar education level. This issue has been acknowledged in other realms of research such as Autism, where children with autism have a “slower” cognitive processing speed when measured with paper-and-pencil tasks, but not when measured with psychophysical IT tasks (Scheuffgen et al., 2000). Given that IT enables a non-motor rapid and accurate assessment of the speed of visual processing and discrimination of a visual stimulus, our results suggest that the IT task would be a quick and effective addition to cognitive measures in many clinical health related disciplines. Thus, the implications of the results are important to aging research, and especially for use with clinical populations where motor speed may be severely compromised, i.e., in stroke.

## Ethics statement

This study was carried out in accordance with the recommendations of National Statement on Ethical Conduct in Human Research with written informed consent from all subjects. All subjects gave written informed consent in accordance with the Declaration of Helsinki. The protocol was approved by University Human Ethics Committee (UHEC), La Trobe University.

## Author contributions

SC initiated the project, designed the outline and content and has contributed to the writing. DE has contributed to most of the writing, and developed the first and final draft of the manuscript. KM has contributed to some of the writing. DE, KM, and DC largely contributed to participant recruitment, data collection and data analysis. AB developed the modified Inspection Time task, and provided clear protocols on how to conduct the task with participants. AB also ran practice trials using the task with all researchers, which in turn assisted in data collection.

## Funding

The project has been approved by La Trobe University Human Ethics Committee (UHEC), approval number S15/19. All funding has been provided by La Trobe University, School of Psychology and Public Health, Department of Psychology and Counselling.

### Conflict of interest statement

The authors declare that the research was conducted in the absence of any commercial or financial relationships that could be construed as a potential conflict of interest. The reviewer JH and handling Editor declared their shared aý liation, and the handling Editor states that the process nevertheless met the standards of a fair and objective review.
